# Demographic characteristics of an avian predator, Louisiana Waterthrush (*Parkesia motacilla*), in response to its aquatic prey in a Central Appalachian USA watershed impacted by shale gas development

**DOI:** 10.1371/journal.pone.0206077

**Published:** 2018-11-28

**Authors:** Mack W. Frantz, Petra B. Wood, George T. Merovich

**Affiliations:** 1 West Virginia Cooperative Fish and Wildlife Research Unit, Davis College, School of Natural Resources, West Virginia University, Morgantown, WV, United States of America; 2 U.S. Geological Survey, West Virginia Cooperative Fish and Wildlife Research Unit, West Virginia University, Morgantown, WV, United States of America; 3 Fisheries and Aquatic Sciences Program, Department of Environmental Science, Juniata College, Huntingdon, PA, United States of America; College of Agricultural Sciences, UNITED STATES

## Abstract

We related Louisiana Waterthrush (*Parkesia motacilla*) demographic response and nest survival to benthic macroinvertebrate aquatic prey and to shale gas development parameters using models that accounted for both spatial and non-spatial sources of variability in a Central Appalachian USA watershed. In 2013, aquatic prey density and pollution intolerant genera (i.e., pollution tolerance value <4) decreased statistically with increased waterthrush territory length but not in 2014 when territory densities were lower. In general, most demographic responses to aquatic prey were variable and negatively related to aquatic prey in 2013 but positively related in 2014. Competing aquatic prey covariate models to explain nest survival were not statistically significant but differed annually and in general reversed from negative to positive influence on daily survival rate. Potential hydraulic fracturing runoff decreased nest survival both years and was statistically significant in 2014. The EPA Rapid Bioassessment protocol (EPA) and Habitat Suitability Index (HSI) designed for assessing suitability requirements for waterthrush were positively linked to aquatic prey where higher scores increased aquatic prey metrics, but EPA was more strongly linked than HSI and varied annually. While potential hydraulic fracturing runoff in 2013 may have increased Ephemeroptera, Plecoptera, and Trichoptera (EPT) richness, in 2014 shale gas territory disturbance decreased EPT richness. In 2014, intolerant genera decreased at the territory and nest level with increased shale gas disturbance suggesting the potential for localized negative effects on waterthrush. Loss of food resources does not seem directly or solely responsible for demographic declines where waterthrush likely were able to meet their foraging needs. However collective evidence suggests there may be a shale gas disturbance threshold at which waterthrush respond negatively to aquatic prey community changes. Density-dependent regulation of their ability to adapt to environmental change through acquisition of additional resources may also alter demographic response.

## 1. Introduction

The rapid development of hydraulic fracturing techniques in the last decade has allowed the expansion of development for unconventional drilling activity, hereafter shale gas development [1]. The Marcellus-Utica shale basin is one of the largest natural gas plays underlying part of the northeastern United States with substantial growth in gas production [2−3]. As of 2015, over 140,000 ha of land have been developed, with deciduous forest one of the major habitat types affected with high ecosystem service costs [4]. Shale gas development has outpaced the ability to create adequate management practices that protect against harm to aquatic and terrestrial wildlife communities and their habitat [5]. The trend for core forest disturbance from shale gas development where headwater streams occur [6] stresses the need for regional monitoring and research in these ecosystems.

Although there is local and regional variability in risks to water resources from shale gas development [7], shale gas development commonly occurs <300m from streams, increasing the threat of surface water degradation from sedimentation, altered stream flow, and the introduction of contaminants [8]. Johnson et al. [9] found that differences in benthic macroinvertebrate communities were dependent on the level of gas activity, and Grant et al. [10] found that stream pH, fish biodiversity, and taxa richness were negatively correlated with the number of gas wells. Additionally, Lutz and Grant [11] found that shale gas disturbed streams were more acidic and had lower index of biotic integrity (IBI) scores. However, other studies found shale gas development did not have any noticeable impact on water quality [12], or in least intrusive scenarios no evidence of impacts on fish, salamander, and crayfish assemblages [13]. Shale gas development has the potential to alter the base of aquatic food webs [14] and may be associated with bioaccumulated contaminants in an apex predator [15], but no study has yet followed potential effects from shale gas development across trophic levels of the aquatic-terrestrial interface.

Terrestrial and aquatic ecosystems are closely linked through cross-habitat physical mechanisms and energy fluxes, leaving research focusing only on land or water ecologically incomplete [16]. In particular, dynamics of forested headwater stream ecosystems occur at the aquatic-terrestrial interface [17]. Headwater streams are the critical sources of water, sediment, organic matter, and nutrients for the rest of the system [18], and are therefore vital for ecological integrity [19]. Furthermore, headwater streams, despite their predominance of drainage area and total stream length, are largely overlooked for protection or regulation despite their strong influence on downstream reaches [20].

Species with specialized terrestrial or aquatic habitat needs that overlap forested freshwater ecosystems [21−22] undergoing shale gas development may be the most vulnerable to disturbance [5]. The Louisiana Waterthrush (*Parkesia motacilla*), hereafter waterthrush, is a habitat specialist and species of conservation concern [23] that breeds in contiguous riparian forests [24] and forages on benthic macroinvertebrates, hereafter aquatic prey, in well-developed riffle and pool areas [25]. Waterthrushes are considered bioindicators of riparian ecosystem integrity [25] due to their stream dependency [26–27].

Over a six-year waterthrush demography study (2009–2011, 2013–2015) at Lewis Wetzel Wildlife Management Area (LWWMA) located in northwestern West Virginia, we observed general annual declines in territory density, reproductive success, and riparian habitat quality with increases in shale gas development [28], as well as declines in site fidelity and apparent survival (M. Frantz, *pers*. *comm*.). In 2011 and 2013–2014, two benthic studies on the same study area linked shale gas development to both strong (2011) and weak (2013–2014) negative influences on benthic community structure ([29]; G. Merovich, *pers*. *comm*.). The 2011 study also evaluated waterthrush demographic response to aquatic prey and found territory density and clutch size were greater in higher quality stream corridors during a year when shale gas activity was high [29]. However, Wood et al. [29] spanned only one breeding season with a limited sample size (n = 12 watershed samples) at the watershed-scale, meriting further evaluation with increased sampling efforts at other spatial scales (territory and nest).

As a follow-up to these previous studies, in 2013–2014 we: 1) evaluated the congruence between aquatic prey and riparian quality indices used to gauge waterthrush habitat (i.e., US EPA Rapid Bioassessment Protocol, EPA; Habitat Suitability Index, HSI), 2) evaluated if the amount of shale gas disturbance or potential hydraulic fracturing runoff in a territory or at a nest influence aquatic prey, and 3) quantified waterthrush demographic response to aquatic prey changes. We hypothesized that aquatic prey should be positively linked with riparian quality habitat scores. EPA and HSI scores were negatively affected by shale gas development [28]. As a consequence of habitat degradation, we expected a negative relationship between aquatic prey metrics and the amount of shale gas disturbance or potential hydraulic fracturing runoff in a territory or at a nest. We also hypothesized that clutch size, number of fledglings, and territory density would have a positive association with aquatic prey metrics. Annual territory length increased as territory densities decreased [28], so we expected smaller territories to be indicative of higher quality aquatic prey and stream quality (e.g., [30]). Nest survival was minimally affected by aquatic prey in 2011 [29] but we hypothesized that any stream impairment effects on the aquatic prey would affect nest survival.

## 2. Methods

### 2.1 Study area

**Fig 1:**
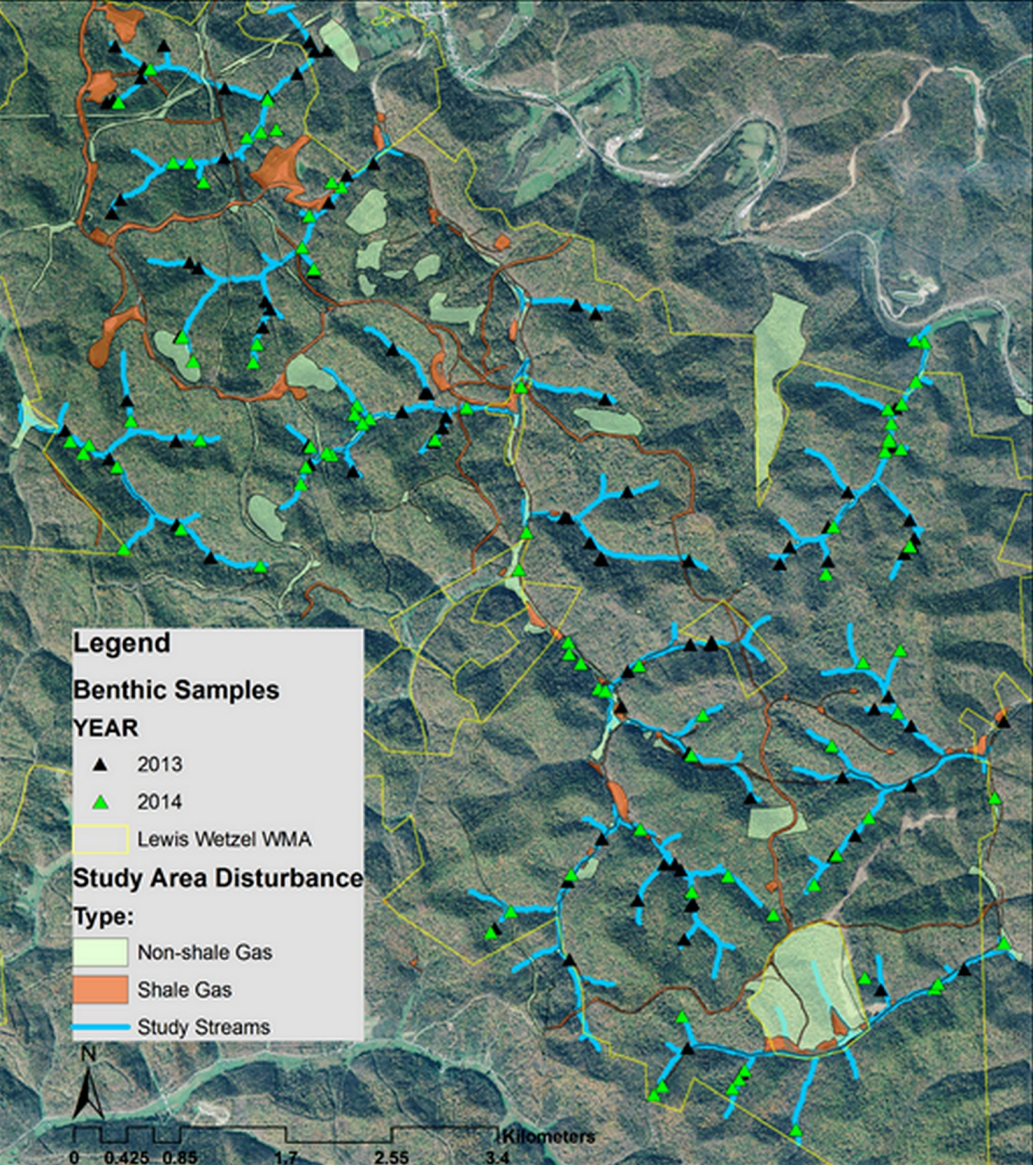
Location of study streams, benthic sampling locations, and stream reaches disturbed by shale gas development during 2013–2014 on the Lewis Wetzel Wildlife Management Area in northwestern West Virginia. The larger light green patches of non-shale gas disturbance are primarily timber harvests with partial canopy removal.

We studied waterthrush along 58.1 km of 1st- and 2nd-order headwater stream tributaries (n = 14) at Lewis Wetzel Wildlife Management Area (LWWMA) located in northwestern West Virginia (Fig 1). Our waterthrush aquatic prey study in 2013–2014 was part of a waterthrush demography study over a six year period (2009–2011, 2013–2015; [28]). The study area overlays the Marcellus-Utica shale region and occurs where waterthrush reach their highest densities within the central Appalachians [31]. The LWWMA is part of a regional core designated as a priority conservation planning area for both aquatic and terrestrial targets [32].

During our study, shale gas development activities included building of conventional (shallower formations) and Marcellus well pads, timbering for yet unbuilt well pads, the expansion of existing road and pipeline infrastructure, and the construction of new infrastructure. In 2008, the LWWMA was 95.3% forested and had 0.4% shale gas land cover; the first shale gas well development began in 2007 [33]. Between the 2010 and 2011 breeding seasons, shale gas development activities that occurred since 2007 accelerated across the study area and began to increase especially on ridgetops. In 2011, study area-wide shale gas land cover was 1.3% and increased to 2.7% in 2013–2014 ([33]; Table 1). Starting in 2013, shale gas development abated study area-wide and instead became concentrated to specific streams and ridgetops. Clearing for additional new well pads occurred late (June–July) in the 2013 breeding season with well pad completion in 2014, in addition to re-drilling of an existing well pad. There was no new shale gas development or activity in the 2015 breeding season. In 2015, the LWWMA was 90.8% forested and 3.9% in shale gas development land cover, with 83.1% of shale gas development resulting in direct forest loss [33]. In summary, 2013 disturbances slowed and affected streams more noticeably late in the breeding season, while in 2014 shale gas activity peaked again, particularly at Slabcamp Run, but did not achieve 2011 levels of activity (S1 Table).

**Table 1:** Louisiana Waterthrush annual demographic, riparian habitat quality, and shale gas disturbance metrics (mean ± SE) at Lewis Wetzel Wildlife Management Area, WV at peak (2011) and later stages (2013–2014) of shale gas development. Our study associated waterthrush response to aquatic prey community changes in relation to shale gas disturbance. All metrics are a subset of those originally reported in Frantz et al. [28] excepting % shale gas land cover which is cited from Farwell et al. [33]. Variable names are defined in S2 Table.

Variable	2011	2013	2014
**Riparian Habitat Quality**			
EPA Index (range 0–200)	158.6 ± 1.8	148.9 ± 2.1	165.6 ± 2.2
HSI (range 0–1)	0.78 ± 0.02	0.76 ± 0.02	0.77 ± 0.02
**Demography**			
Territory Density	1.5 ± 0.1	1.2 ± 0.1	1.1 ± 0.1
Territory Length (m)	556.4 ± 31.2	659.0 ± 34.3	772.1 ± 41.9
Nest Survival	38.0 ± 8.0	28.5 ± 6.1	25.7 ± 5.8
Clutch Size	4.8 ± 0.1	4.6 ± 0.1	4.4 ± 0.1
Fledglings	4.5 ± 0.1	4.7 ± 0.1	4.1 ± 0.2
**Disturbance**			
% TerrGas	38.0 ± 5.2	18.0 ± 3.4	27.2 ± 4.5
% TerrRunoff	20.0 ± 4.5	32.9 ± 5.2	36.0 ± 5.0
% StreamGas	32.3 ± 6.3	17.3 ± 5.7	21.5 ± 6.4
% StreamRunoff_	19.3 ± 5.7	24.2 ± 5.5	24.2 ± 5.5
% Shale Gas Land Cover	1.3	2.7	2.7

### 2.2 Mapping of streams and shale gas disturbance

Within a Geographic Information System (GIS), we used a sequence of leaf-on and leaf-off aerial photographs from the National Agriculture Imagery Program (NAIP) for 2011 and 2014, satellite Quickbird imagery for 2009, and extensive annual ground-truthing to manually digitize areas of disturbance within the study area for each year of the long-term study, including years 2013–2014 of the aquatic prey study (see Frantz et al. [28] for full description). All forest canopy disturbances were classified as shale gas related (i.e., well pads and associated road and pipeline infrastructure, frequent truck traffic, and visual stream sedimentation) or as being unrelated or pre-existing (i.e., forest roads, recent even-aged timber harvests, and various types of existing clearings). We classified a few conventional impacts (i.e., stream-side vertical pump jacks) as related to shale gas development because their pads were managed in conjunction with nearby shale gas infrastructure and because their targeted formation, even though they remained shallow after development, was listed as Marcellus [34]. Gas well records [35] were used to verify target shale formations, drilling status, and start dates for all well disturbances.

Lengths of each study stream (average length 4.1 ± 0.54 km, range 0.95–7.4 km) were calculated in GIS using a 3D functional surface length tool and a 3 m resolution digital elevation model to account for topography, and study streams were defined to have a drainage basin of 9.0 hectares (i.e., <100 ha; [36]) to delineate the uppermost headwater reaches (24 k scale or higher resolution; e.g., [37]). To describe and model waterthrush demography and riparian habitat quality as a function of shale gas disturbance, we created four continuous and one binary variable based on disturbance categories at the stream, territory, and nest scale. The first (termed StreamGas) described mostly localized streamside disturbance indicative of the presence of any shale gas infrastructure or activity. A section of stream was considered disturbed when well pads, infrastructure, or frequent vehicular activity were within 60 m of the stream centerline, which is the typical extent of waterthrush streamside use (i.e., 60 m; [38]). When a stream had visually observable sedimentation that resulted from shale gas development at any distance from the stream, we classified the entire stream network downstream of the sedimentation beginning point as disturbed. Streams were frequently and extensively ground-truthed each season, so there were no stream reaches where sedimentation events were likely to be missed.

We created a second shale gas disturbance category (termed StreamRunoff) that focused solely on potential run-off into streams from shale gas contaminants. A stream was considered disturbed from at and below a well pad or retaining pond (similar to Latta et al. [15]), resulting in the whole downstream network classified as at risk for surface pollution based on elevational maps and ground truthing. This category did not include pipeline or road disturbance and was a broader, distance-independent, disturbance category describing potential water pollution. For each year of the study, we then calculated the proportion of each stream disturbed for each of these two disturbance categories.

We calculated the proportion of each waterthrush territory (a 60-m buffer around each territory vector) that was disturbed by StreamGas and called this metric TerrGas. The proportion of each territory disturbed by StreamRunoff was termed TerrRunoff. We classified each waterthrush nest location as undisturbed or disturbed by StreamGas within 60-m around the nest and called this variable NestGas. Hereafter we use StreamGas, StreamRunoff, TerrGas, TerrRunoff, and NestGas to describe shale gas disturbance metrics (Table 1, S2 Table).

### 2.3 Waterthrush riparian habitat quality

Riparian habitat quality was assessed using the Habitat Suitability Index specifically designed for waterthrush (hereafter HSI; [25]) and the US EPA Rapid Bioassessment Protocol for high gradient streams (hereafter EPA [39]) in the same manner as Wood et al. [29] and Frantz et al. [28]. The HSI is a broad-scale evaluation of waterthrush instream foraging habitat, nesting habitat, and upland habitat suitability [25]. The EPA assesses stream quality based primarily on instream characteristics that relate to the abundance and composition of aquatic organisms, and therefore may indicate relative quality of instream foraging habitat for waterthrush [29]. The HSI and EPA indices were quantified in a 50-m stream reach centered on each nest location monitored to make the indices sensitive to habitat immediately surrounding waterthrush nests.

### 2.4 Waterthrush demographic monitoring

We quantified annual waterthrush territory length (m), territory density (# territories/km), and nest survival for our 14 study streams as described in Frantz et al. [28]. Waterthrush territories were delineated as linear vectors along each stream during April 1–June 29 using standardized territory mapping (≥6 stream visits [40–41]). Nest searching and monitoring occurred concurrently with territory mapping. Locations of waterthrush observations and nests were recorded with a WAAS-enabled Garmin 60CSX GPS unit with accuracy ≤5 m in 2013–2015.

To calculate daily survival rate (DSR) for nest survival, we monitored nests typically every three-four days initially and more frequently as fledging approached [42]. We assumed an undamaged empty nest had fledged if the nest was active the day before and had approached the predicted fledge date. Nest sites were revisited at least one more time to verify either no activity or renesting if the nest was not active prior to the expected fledge date. We counted number of eggs to determine clutch size of nests with complete clutches, and the number of fledglings for each successful nest was the count of nestlings in the visit prior to fledging. Nests were considered successful if they produced at least one waterthrush fledgling, including nests parasitized by Brown-headed Cowbirds (*Molothrus ater*).

### 2.5 Aquatic prey sampling

Aquatic prey occurring in riffle habitat adjacent to nest site locations were sampled once per nest using a Surber sampler. Nest site samples (n = 178) were collected shortly after the nest fledged, failed, or had been abandoned (May 22 –July 28, 2013; June 16 –July 6, 2014) to assess relative prey availability near the time a nest contained fledglings. During sample collection, we scrubbed rocks (>8 cm in diameter) and disturbed sediment 3-cm below the stream bed within the Surber frame for a total of 3 minutes [27]. We separated aquatic prey from detritus for each sample in the field and stored all organic matter in 70% or 95% ethanol.

Post-field season, aquatic prey in benthic samples were sorted, counted, and identified to genus level. Body lengths were also measured to estimate biomass (crayfish excluded). To summarize the aquatic prey composition for each sample, we calculated a family level multimetric IBI called the West Virginia Stream Condition Index (WVSCI [43]), and a genus level multimetric IBI called the Genus Level Index of Most Probable Stream Status (GLIMPSS), version CF, which does not require the genus-level identification of Chironomidae or Oligochaeta [44]. The values we calculated for both indices are based on sampling methods that are slightly modified [45] from the standard methods (i.e., Surber samples and all individuals used in calculations). Thus, they are not strictly interpretable as indicators of stream ecosystem health as originally intended. Nevertheless, they still quantify the composition and integrity of the aquatic prey resource available to waterthrush. We additionally calculated overall aquatic prey density and biomass using length-mass regressions [46]. In total, we selected six aquatic prey metrics to relate to waterthrush demography: WVSCI, GLIMPSS, biomass, density, EPT richness (component of WVSCI), and number of intolerant genera (component of GLIMPSS where pollution tolerance value is <4); S2 Table).

### 2.6 Analysis

We used spatial generalized linear mixed models (hereafter SGLMMs) to assess relationships between waterthrush demography and aquatic prey as well as between riparian habitat quality and aquatic prey for each year (i.e., 2013, 2014) separately. SGLMMs accounted for possible effects of spatial autocorrelation and were modeled using *corrHLfit* within the spaMM package [47–48] in R [49]. Model residuals were evaluated graphically, extreme or influential data outliers identified graphically and with packages car [50] and stats [49] for potential removal, and other data exploration diagnostic tools were used [51] to ensure model assumptions were met. We used x-y coordinates as a spatial random effect in a Matern correlation model and included a stream random effect. For all SGLMMs, we determined statistical significance of fixed effects using a likelihood ratio test and set significance at α = 0.10 to be cautiously moderate in our assessment of biological significance [52].

We evaluated the degree to which the six aquatic prey metrics were related to riparian habitat quality (i.e., EPA and HSI scores) in individual SGLMM models. We also tested the relationship between the six aquatic prey metrics and the shale gas disturbance metrics for the nest and territory scales (TerrGas, TerrRunoff, and NestGas) as G. Merovich (*pers*. *comm*.) found differences in benthic communities up and downstream of shale gas development. We modeled WVSCI, GLIMPSS, biomass, and density using a Gaussian distribution with biomass and density receiving a log10 transformation to approximate normality. We removed an outlier from our benthic density dataset because it was identified as a strong influential outlier not representative of other samples (6422.2 m^2^ vs. 354.7 ± 31.3 per m^2^ average density) by using the outlierTest and influence.measures functions with packages car and stat. EPT richness and number of intolerant genera were modeled using the Conway-Maxwell-Poisson (COM-Poisson [53–54]) distribution that generalizes the Poisson distribution to handle a wide range of under and over-dispersion typically found in ecological count data [55]. If a COM-Poisson model could not converge, we substituted with a Poisson distributed model (n = 12 models). Because Wood et al. [29] did not assess EPA and HSI in relation to aquatic prey metrics for their 2011 data, we completed a retrospective analysis of their 2011 data. We used Pearson (i.e., EPA) and Spearman (i.e., HSI) correlation coefficients to relate 2011 EPA and HSI to aquatic prey metrics with package psych [56] for comparison to our 2013–2014 results.

We additionally assessed the relationship between waterthrush demography (number of fledglings, clutch size, territory length, and territory density) and the six aquatic prey variables in individual SGLMM models as above. Number of fledglings, clutch size, and territory density were modeled using the COM-Poisson distribution. Territory length was modeled using a gamma distribution. We removed an outlier sample from the fledging dataset where only one fledgling was represented due to Brown-headed Cowbird (*Molothrus ater*) parasitism. Because the Wood et al. [29] aquatic prey study did not assess territory length, we used a Pearson correlation on data collected in 2011 in the same manner as riparian habitat quality above to relate the six aquatic prey variables to territory length for comparison to our 2013–2014 results.

We used program MARK 7.1 (Colorado State University, Ft. Collins, Colorado, USA, [57]) to estimate daily survival rate (DSR) of monitored waterthrush nests in 2013 and 2014. We removed 63 nests that did not meet the assumption requirements of program MARK and 11 nests that had no benthic information, leaving 107 nests for analysis. We assumed a 29-day nesting period (egg-laying 5, incubation 14, nestling 10 days) based on the chronology of nests monitored on our study area [28].

We developed a set of 7 *a priori* candidate models [58] that we hypothesized might influence DSR of waterthrush nests based on the results of Wood et al. [29] and Frantz et al. [28]. All covariates are defined in S2 Table. All *a priori* models included 3 temporal covariates and a shale gas covariate that influenced nest survival in our study area [28]; they included nest age (NestAge), quadratic effect of time of nesting within the breeding season (TT), average daily rainfall (Rain), and percent potential hydraulic fracturing runoff within a territory (TerrRunoff). Instead of an intercept model with no covariates, these 4 covariates formed our base null model given their known importance [28], allowing us to assess whether aquatic prey also influenced nest survival by accounting for them. Nest age indicates vulnerability as the nest ages [59] and within-season trends in DSR reflect dynamic activity patterns of nest predators (e.g., [60]). Mean daily rainfall (in mm) by influencing prey availability affects waterthrush nest survival [28, 38] as headwater riparian systems are subject to seasonality and annual changes in rainfall [61]. For each nest, we averaged daily rainfall estimates across the period in which an active nest was under observation [38]. Precipitation estimates were pooled from four Weather Underground, Inc. network stations closest (avg. 36 km) to the study area. Six additive models included the null model plus each of our aquatic prey covariates of interest.

We used Akaike’s Information Criterion for small sample sizes (AICc) to evaluate support for candidate models [62] in program MARK. We modeled the binomially distributed data with the user-defined, logit-link function while simultaneously considering associations with the covariates of interest. We considered the model with the lowest AICc value to be the best-supported model given the data, and any models with ΔAICc <2 were considered plausible. We used regression coefficients and 85% confidence intervals (hereafter CIs) to infer biological importance of covariates in plausible AICc models [63]. We model-averaged NestAge, TT, Rain, and TerrRunoff across all models [62].

## 3. Results

**Fig 2:**
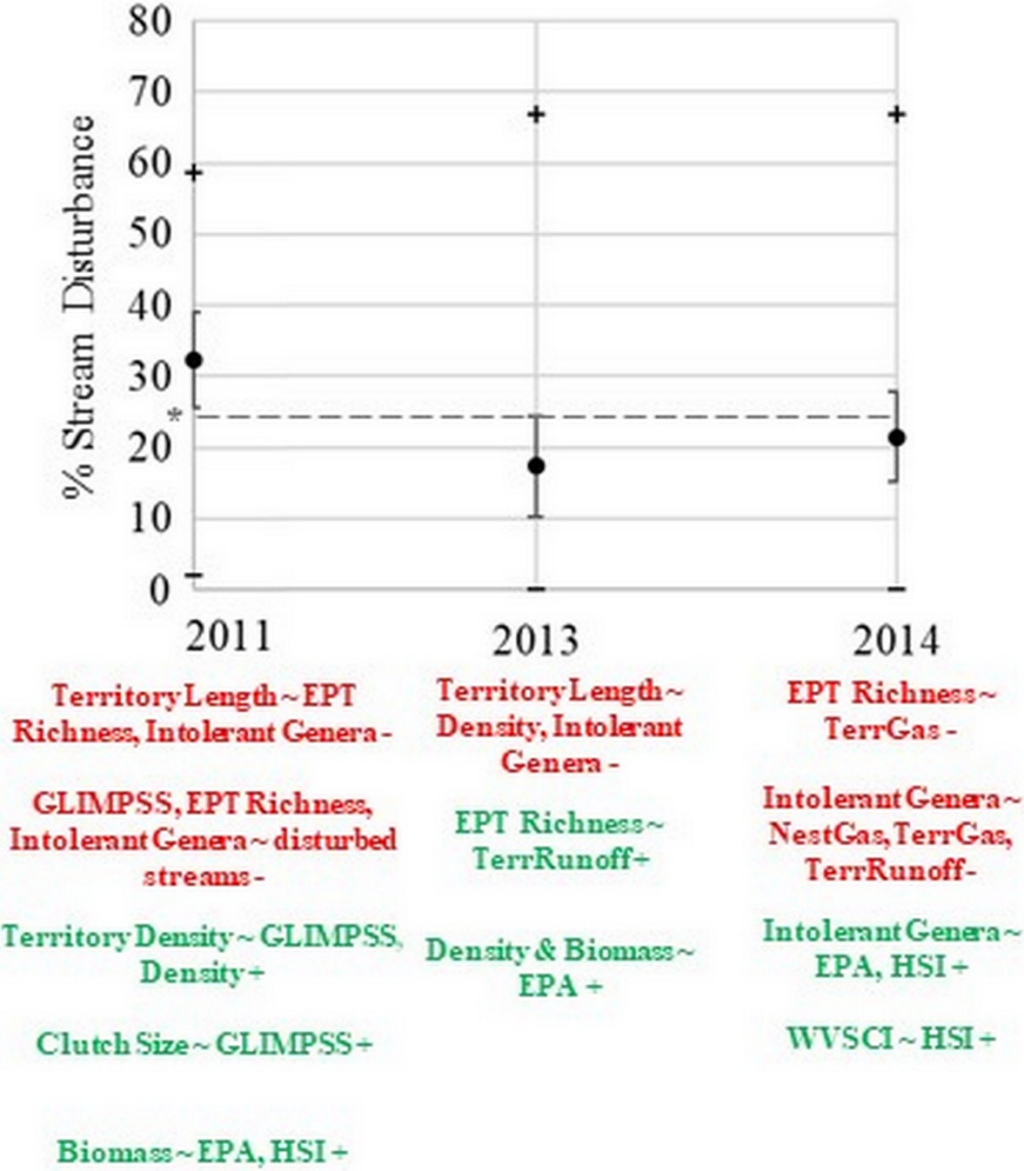
The average amount of shale gas related disturbance ± standard error (SE) and range (black + and -) on headwater streams (n = 14), in addition to statistically significant positive (green) and negative (red) demographic vs. aquatic prey responses over a six year period (2009–2011, 2013–2015) at Lewis Wetzel Wildlife Management Area (LWWMA) located in northwestern West Virginia. Nest survival results are not displayed. The bracketed line represents a hypothetical, conservative disturbance threshold (≥25%) at which waterthrush demography may be more negatively affected based on the strongest and second strongest demographic responses to aquatic prey in 2011 and 2014. Variable names are defined in S2 Table.

Stream disturbance due to shale gas (i.e., StreamGas) was 32.3% in 2011, dropped to 17.3% in 2013, and elevated to 21.5% in 2014, reflecting different levels of shale gas activity despite study area-wide shale gas land cover not changing between 2013 and 2014 (Table 1; S1 Table; Fig 2). The potential for hydraulic fracturing runoff within streams (i.e., StreamRunoff) increased from 19.3% in 2011 to 24.2% in 2013 and 2014 (Table 1). The percent of each waterthrush territory disturbed by shale gas (i.e., TerrGas) had the same patterns as StreamGas while TerrRunoff increased each year (Table 1). Only 20.3% of territories (39 of 192 total from 2011, 2013–2014) had their full territory length (100%) disturbed by TerrGas or TerrRunoff.

In 2013, aquatic prey biomass and density increased with increasing EPA score, while in 2014 intolerant genera increased with increasing EPA score (Table 2; Fig 2). No relationships were statistically significant in 2013 between HSI and aquatic prey, but in 2014 intolerant genera and WVSCI (approaching significance) increased with increasing HSI score (Table 2; Fig 2). For 2011 data, aquatic prey biomass had a statistically significant, positive correlation with EPA (R^2^ = 0.67, *P* = 0.02) and HSI (Rho = 0.51, *P* = 0.09). In 2013, EPT richness increased with increasing TerrRunoff, but in 2014 EPT richness decreased with increasing TerrGas (Table 3; Fig 2). In 2014, intolerant genera decreased with increasing TerrRunoff, TerrGas, and NestGas (Table 3; Fig 2). Raw data used to analyze the relationship between riparian habitat quality and aquatic prey can be found in S1 Dataset (years 2013–2014) and S2 Dataset (year 2011), and S3 Dataset for the relationship between shale gas disturbance and aquatic prey.

**Table 2:** Association between waterthrush riparian habitat quality indices (i.e., EPA and HSI) and aquatic prey metrics in spatial generalized linear mixed models. In 2013, aquatic prey biomass and density increased with increasing EPA score, while in 2014 intolerant genera increased with increasing EPA score. No relationships were statistically significant in 2013 between HSI and aquatic prey, but in 2014 intolerant genera and WVSCI (approaching significance) increased with increasing HSI score. Results with ^P^ are from a Poisson model. P values of variables that are statistically significant are bolded. Variable names are defined in S2 Table. LRT = likelihood ratio test χ2 statistic. β = beta estimate of fixed effect.

Independent Variable	β ± SE	LRT χ^2^	p value	β ± SE	LRT χ^2^	p value
	**GLIMPSS**			**WVSCI**		
**Year 2013**						
EPA	0.120 ± 0.092	1.670	0.196	0.006 ± 0.081	0.010	0.922
HSI	13.700 ± 14.480	0.938	0.333	1.991 ± 12.515	0.030	0.864
**Year 2014**						
EPA	0.069 ± 0.066	1.128	0.288	0.014 ± 0.045	0.464	0.496
HSI	10.890 ± 11.221	0.961	0.327	11.540 ± 7.582	2.594	**0.107**
	**Density**			**Biomass**		
**Year 2013**						
EPA	0.005 ± 0.002	5.000	**0.025**	0.010 ± 0.004	2.862	**0.091**
HSI	-0.307 ± 0.399	0.601	0.438	0.219 ± 0.752	0.106	0.744
**Year 2014**						
EPA	0.0003 ± 0.002	0.017	0.896	0.002 ± 0.003	0.771	0.380
HSI	0.337 ± 0.351	0.645	0.422	0.148 ± 0.494	0.171	0.679
	**EPT Richness**			**Intolerant Genera**		
**Year 2013**						
EPA	0.010 ± 0.015	0.599	0.439	0.006 ± 0.005 ^P^	1.665 ^P^	0.197 ^P^
HSI	-1.026 ± 2.517	0.036	0.850	-0.679 ± 0.718 ^P^	0.869 ^P^	0.351 ^P^
**Year 2014**						
EPA	-0.005 ± 0.007	0.327	0.567	0.005 ± 0.002	3.160	**0.075**
HSI	1.581 ± 1.266	2.109	0.146	0.828 ± 0.399	4.573	**0.032**

**Table 3:** Association between waterthrush aquatic prey and shale gas disturbance metrics in spatial generalized linear mixed models. Results with ^P^ are from a Poisson model. In 2013, EPT richness increased with increasing TerrRunoff, but in 2014 EPT richness decreased with increasing TerrGas. In 2014, intolerant genera decreased with increasing TerrRunoff, TerrGas, and NestGas. P values of variables that are statistically significant are bolded. Variable names are defined in S2 Table. LRT = likelihood ratio test χ2 statistic. β = beta estimate of fixed effect.

Independent Variable	β ± SE	LRT χ^2^	p value	β ± SE	LRT χ^2^	p value
	**GLIMPSS**			**WVSCI**		
**Year 2013**						
TerrGas	-0.008 ± 0.062	0.020	0.888	0.012 ± 0.055	0.053	0.818
TerrRunoff	0.024 ± 0.044	0.253	0.615	0.046 ± 0.039	1.372	0.241
NestGas	0.303 ± 3.745	0.003	0.958	-1.028 ± 3.048	0.112	0.738
**Year 2014**						
TerrGas	-0.054 ± 0.046	1.398	0.237	-0.022 ± 0.033	0.391	0.532
TerrRunoff	-0.029 ± 0.035	0.622	0.430	-0.026 ± 0.025	1.640	0.200
NestGas	-1.989 ± 3.270	0.367	0.545	-0.748 ± 2.277	0.100	0.752
	**Density**			**Biomass**		
**Year 2013**						
TerrGas	0.002 ± 0.002	2.388	0.122	0.005 ± 0.003	2.338	0.126
TerrRunoff	0.002 ± 0.001	2.162	0.141	0.003 ± 0.002	0.469	0.493
NestGas	0.044 ± 0.095	0.219	0.640	0.215 ± 0.179	1.495	0.221
**Year 2014**						
TerrGas	-0.0004 ± 0.001	0.040	0.842	-0.00004 ± 0.002	0.003	0.960
TerrRunoff	-0.0002 ± 0.001	0.006	0.939	0.0004 ± 0.002	0.085	0.771
NestGas	-0.061 ± 0.098	0.280	0.597	0.003 ± 0.144	0.006	0.940
	**EPT Richness**			**Intolerant Genera**		
**Year 2013**						
TerrGas	0.003 ± 0.003 ^P^	0.576 ^P^	0.448 ^P^	0.012 ± 0.012	1.071	0.301
TerrRunoff	0.017 ± 0.008	4.381	**0.036**	0.007 ± 0.008	0.789	0.375
NestGas	-0.034 ± 0.175	0.068	0.794	0.215 ± 0.672	0.114	0.736
**Year 2014**						
TerrGas	-0.010 ± 0.006	2.572	**0.109**	-0.004 ± 0.002	4.934	**0.026**
TerrRunoff	-0.003 ± 0.004	0.681	0.409	-0.003 ± 0.001	4.136	**0.042**
NestGas	-0.424 ± 0.399	1.056	0.304	-0.180 ± 0.112	2.756	**0.097**

All tests for the relationships between clutch size, number of fledglings, and territory density with aquatic prey metrics were statistically non-significant (Table 4). Territory length decreased with increasing aquatic prey density and number of intolerant genera in 2013 (Table 4; Fig 2). For 2011 data, territory length had a statistically significant, negative correlation with GLIMPSS, EPT richness, and number of intolerant genera (R^2^ = -0.65, -0.68, -0.67, *P* = 0.02; Fig 2), respectively. Raw data used to analyze the relationships between waterthrush demography and aquatic prey can be found in S4 Dataset.

**Table 4:** Association between waterthrush demographic response (i.e., clutch size, number of fledglings, territory length and territory density) and its aquatic prey in spatial generalized linear mixed models. All tests for the relationships between clutch size, number of fledglings, and territory density with aquatic prey metrics were statistically non-significant. Territory length decreased with increasing aquatic prey density and number of intolerant genera in 2013. Results with ^P^ are from a Poisson model. P values of variables that are statistically significant are bolded. Variable names are defined in S2 Table. LRT = likelihood ratio test χ2 statistic. β = beta estimate of fixed effect.

Dependent Variable	β ± SE	LRT χ^2^	p value	β ± SE	LRT χ2	p value
	**GLIMPSS**			**WVSCI**		
**Year 2013**						
Clutch size	-0.009 ± 0.012	0.535	0.464	-0.004 ± 0.013	0.100	0.751
Fledglings	-0.004 ± 0.017	0.056	0.812	0.003 ± 0.019	0.831	0.362
Territory length	0.001 ± 0.001	0.143	0.705	-0.001 ± 0.003	-0.790	1.000
Territory density	-0.0003 ± 0.009	0.001	0.970	-0.002 ± 0.004 ^P^	0.445 ^P^	0.505 ^P^
**Year 2014**						
Clutch size	0.002 ± 0.014	0.016	0.900	0.017 ± 0.020	0.734	0.392
Fledglings	-0.019 ± 0.026	0.523	0.469	-0.007 ± 0.041	0.033	0.859
Territory length	0.001 ± 0.001	0.341	0.559	0.002 ± 0.003	0.745	0.388
Territory density	0.001 ± 0.004 ^P^	0.037 ^P^	0.847 ^P^	0.001 ± 0.012	0.007	0.934
	**Density**			**Biomass**		
**Year 2013**						
Clutch size	-0.00002 ± 0.001^P^	0.001^P^	0.975^P^	0.00004 ± 0.0002^P^	0.047^P^	0.828^P^
Fledglings	0.0001 ± 0.001^P^	0.009^P^	0.924^P^	0.0001 ± 0.001	0.009	0.924
Territory length	-0.001 ± 0.0003	8.535	**0.003**	-0.0003 ± 0.0002	2.338	0.126
Territory density	-0.0001 ± 0.001	0.009	0.925	-0.0001 ± 0.001	0.086	0.769
**Year 2014**						
Clutch size	0.0003 ± 0.0005	0.465	0.495	0.00001 ± 0.0001^P^	0.012^P^	0.912^P^
Fledglings	0.0005 ± 0.001	0.811	0.368	0.0004 ± 0.0003	2.125	0.145
Territory length	0.00002 ± 0.0001	0.098	0.754	0.000002 ± 0.00004	0.001	0.979
Territory density	-0.00002 ± 0.0003	0.014	0.907	-0.00004 ± 0.0002	0.048	0.826
	**EPT Richness**			**Intolerant Genera**		
**Year 2013**						
Clutch size	-0.005 ± 0.031 ^P^	0.027 ^P^	0.868 ^P^	-0.079 ± 0.067	1.380	0.240
Fledglings	0.008 ± 0.047	0.027	0.870	-0.007 ± 0.041^P^	0.031^P^	0.860 ^P^
Territory length	-0.014 ± 0.017	-0.460	1.000	-0.040 ± 0.018	4.62	**0.032**
Territory density	-0.008 ± 0.023 ^P^	0.162 ^P^	0.687 ^P^	-0.001 ± 0.049	0.001	0.981
**Year 2014**						
Clutch size	0.019 ± 0.041 ^P^	0.213 ^P^	0.645 ^P^	0.020 ± 0.074	0.072	0.788
Fledglings	0.076 ± 0.158	0.233	0.629	-0.054 ± 0.115	0.218	0.641
Territory length	0.023 ± 0.014	2.486	0.115	0.010 ± 0.007	1.864	0.172
Territory density	0.003 ± 0.051	0.004	0.947	-0.001 ± 0.038	0.0003	0.985

Of 7 *a priori* nest survival models (Table 5), 6 different models were supported (ΔAICc <2) in 2013 and 2014. The null base model had the most weight in both years (*w*i = 0.25, 0.28). The model with EPT richness had the most weight of the 5 supported aquatic prey models in 2013 (*w*i = 0.17) and GLIMPSS the most in 2014 (*w*i = 0.18). Regression coefficient 85% CIs overlapped zero for all aquatic prey covariates indicating little, no, or highly variable influence on DSR, but the direction of the relationship between nest survival and aquatic prey switched from negative to positive for 5 of the 6 aquatic prey covariates from 2013 to 2014 (Table 6). In the null base model Rain had positive influence on DSR in 2013 and 2014, while TerrRunoff had negative influence on nest survival in 2014 (Table 6). MARK-formatted files (.inp file extension) used to analyze the relationship between waterthrush nest survival and aquatic prey are the S5 Dataset and S6 Dataset.

**Table 5:** Year 2013 and 2014 AICc model results of 7 *a priori* nest survival models with aquatic prey covariates using Program MARK. Of 7 a priori nest survival models, 6 different models were supported (ΔAICc <2) in 2013 and 2014 with the null base model having the most weight in both years (wi = 0.25, 0.28). ΔAICc = distance from the top model, and *w*_i_ = Akaike weight. Variable names are defined in S2 Table.

Model	AICc	ΔAICc	*w*i
**Year 2013**			
Rain + NestAge + TT + TerrRunoff	152.33	0	0.25
Rain + NestAge + TT + TerrRunoff + EPT Richness	153.12	0.79	0.17
Rain + NestAge + TT + TerrRunoff + WVSCI	153.36	1.04	0.15
Rain + NestAge + TT + TerrRunoff + Density	153.51	1.18	0.14
Rain + NestAge + TT + TerrRunoff + GLIMPSS	154.00	1.67	0.11
Rain + NestAge + TT + TerrRunoff + Biomass	154.30	1.97	0.09
Rain + NestAge + TT + TerrRunoff + Intolerant Genera	154.35	2.02	0.09
**Year 2014**			
Rain + NestAge + TT + TerrRunoff	164.56	0	0.28
Rain + NestAge + TT + TerrRunoff + GLIMPSS	165.39	0.83	0.18
Rain + NestAge + TT + TerrRunoff + EPT Richness	166.35	1.79	0.11
Rain + NestAge + TT + TerrRunoff + WVSCI	166.36	1.80	0.11
Rain + NestAge + TT + TerrRunoff + Intolerant Genera	166.47	1.92	0.11
Rain + NestAge + TT + TerrRunoff + Density	166.48	1.92	0.11
Rain + NestAge + TT + TerrRunoff + Biomass	166.59	2.03	0.10

**Table 6:** Annual waterthrush nest survival covariates found in the top supported (ΔAICc <2, n = 6) and unsupported (n = 1) AICc models based on regression coefficients, standard error (SE), and 85% confidence intervals. In the null base model Rain had positive influence on daily survival rate (DSR) in 2013 and 2014, while TerrRunoff had negative influence on nest survival in 2014. Significant covariates with non-overlapping confidence intervals are bolded. Covariates included in every model to account for their influence (i.e., Rain, NestAge, TT, and TerrRunoff; [28]) have model-averaged regression coefficients and unconditional SEs. Variable names are defined in S2 Table.

Parameter	Estimate	SE	Confidence Interval
**Year 2013**			
** Rain**	**0.415**	**0.191**	**0.140, 0.690**
TerrRunoff	-0.001	0.002	-0.005, 0.002
NestAge	-0.052	0.043	-0.113, 0.009
TT	0.077	0.155	-0.147, 0.300
EPT Richness	-0.116	0.103	-0.317, 0.085
Density	-0.002	0.002	-0.005, 0.002
Biomass	-0.0002	0.001	-0.002, 0.001
WVSCI	-0.018	0.018	-0.054, 0.018
GLIMPSS	-0.009	0.015	-0.037, 0.020
**Not in top supported:**			
Intolerant Genera	-0.014	0.099	-0.208, 0.180
**Year 2014**			
** Rain**	**0.380**	**0.183**	**0.118, 0.643**
** TerrRunoff**	**-0.005**	**0.002**	**-0.008, -0.002**
NestAge	0.016	0.047	-0.052, 0.084
TT	-0.022	0.080	-0.137, 0.094
EPT Richness	-0.052	0.104	-0.255, 0.151
Density	0.0001	0.0004	-0.001, 0.001
WVSCI	0.012	0.023	-0.034, 0.057
GLIMPSS	0.016	0.015	-0.013, 0.045
Intolerant Genera	0.027	0.076	-0.121, 0.175
**Not in top supported:**			
Biomass	0.00004	0.0003	-0.001, 0.001

## 4. Discussion

Shale gas disturbances on our headwater stream ecosystem varied with the intensity of shale gas development that year ([28]; Table 1; S1 Table). Our follow-up study was able to establish how shale gas alterations to riparian habitat quality and the food web can lead to potential effects at a higher trophic level in an apex predator. By also documenting waterthrush demography decline (Table 1, [28]) and shifts in aquatic prey community structure ([29]; G. Merovich *pers*. *comm*.) due to shale gas development, our study establishes the extent of dependency of waterthrush demographic response and adaptation due to the integrity of ecosystem conditions at the aquatic-terrestrial interface.

### 4.1 Waterthrush foraging resources

Our study builds a connection for decreasing riparian habitat quality due to shale gas altering, at least in part, waterthrush foraging resources. The EPA riparian habitat assessment has been successfully used in other studies in conjunction with waterthrush occupancy to explain biotic integrity [27]. Higher EPA index and HSI scores were indicative of a larger and healthier aquatic prey community in our system although not with all metrics and statistical significance was dependent on year (Table 2). Additionally, EPT richness and intolerant genera were negatively related to shale gas disturbance, mainly in 2014 (Table 3); this was important to establish since waterthrush riparian habitat quality was negatively affected by shale gas [28]. Overall, HSI was less reliable than EPA for describing aquatic prey, which may be due to HSI consisting of not just food (i.e., stream habitat and quality) scores, but also scores tabulated for waterthrush habitat cover, nesting, and a surrounding landscape classifier [25].

### 4.2 Waterthrush demographic responses

Most demographic responses to aquatic prey were variable or statistically non-significant. Even so, general demographic responses were negatively related to aquatic prey in 2013 then shifted to a positive response in 2014 when shale gas disturbance had a stronger negative influence on aquatic prey and nest survival (Table 4, Table 5). On streams acidified by mine drainage, waterthrush establish larger territories and forage on peripheral and novel prey items (e.g., terrestrial salamanders) to acquire sufficient prey resources [31]. We saw a similar effect where territory length increased with measures indicating poorer aquatic prey base (e.g., low EPT genus richness). However, territory length in 2014 tended to increase with increasing aquatic prey metrics, opposite of previous years (Table 4). Waterthrush likely had the ability to compensate for loss of food resources by foraging in undisturbed parts of their territory, in addition to increasing territory length, as only 20.3% of territories had their full territory length disturbed by TerrGas or TerrRunoff. The waterthrush’s compensation ability in combination with the decline in annual territory density likely contributed to the disassociation between territory length and aquatic prey in 2014. In contrast, given the stronger response and higher territory densities in 2011, under normal territory density conditions (≥1.5 territories/km) the hypothesis of smaller territories indicate higher quality habitat and foraging resources [30] likely still holds true.

### 4.3 Shale gas disturbance influences on nest survival and aquatic prey

Models used to explain nest survival were also dependent on year (Table 5) with TerrRunoff significantly decreasing daily survival rate in 2014 (Table 6). Our study aligns with Wood et al. [29] in that aquatic prey likely is less influential on nest survival than temporal effects like rain or shale gas disturbance [28]. While our waterthrush-related shale gas disturbance metrics (i.e., TerrGas, TerrRunoff, and NestGas) suggest conflicting yet overall weak negative effects on aquatic prey (Table 3), aquatic prey community structure at our study area quantified upstream and downstream of shale gas at a subwatershed scale also mirrored shale gas activity: community changes differed the most in 2011 [29], were weaker in 2013, and then slightly stronger in 2014 but not as much as 2011 when shale gas activity was highest (G. Merovich, *pers*. *comm*.; S1 Table).

### 4.4. Implications

The year-to-year waterthrush demographic responses to aquatic prey in our study were not strongly proportional but instead followed relatively weaker patterning. Timing of benthic sampling in 2013 in relation to shale gas activity levels likely in part explain the lack of a clear signal between waterthrush demography and its aquatic prey. New shale gas activity in 2013 was not as evident until near or after sampling late in the breeding season (S1 Table), and shale gas well pad construction and drilling typical of our study site and elsewhere occur in “pulses” [5]. With our sampling design, we were able to detect benthic community responses as stronger in 2014 than 2013 (similar to G. Merovich, *pers*. *comm*.), but increased sampling efforts during appropriate sampling periods may be even more critical for higher food web organisms in overcoming variability of demographic response to shale gas.

Our study, through collective evidence, suggests the potential for localized negative effects to aquatic prey from shale gas development, in particular EPT and intolerant taxa that are believed to be the waterthrush’s preferred prey [24]. Additionally, the shift in demographic response in 2014 when shale gas disturbance had stronger negative effects on aquatic prey and nest survival may suggest a shale gas disturbance threshold ([64]; Fig 2) at which waterthrush respond and adapt to aquatic prey in the same manner aquatic prey community structure concurrently reflected levels of annual disturbance ([29]; G. Merovich, *pers*. *comm*.). Waterthrush are most likely to forage in locations that have higher EPT and intolerant genera [29], making it important to maintain or improve riparian habitat quality linked directly with their aquatic prey. In consideration of population regulatory mechanisms (e.g., [65]) that may influence annual demographic response and adaptability, continued long-term monitoring will be required to discern if a threshold of shale gas disturbance exists that alters aquatic prey communities and, in turn, affects demography of higher-level trophic linkages [66−67]. To some degree, waterthrush in our system appear to have the ability to adapt and meet their foraging needs. However, based on the response in 2011 and 2014 when aquatic prey was the most affected by shale gas, mechanisms used to compensate when stream disturbance is conservatively ≥ 25% (Fig 2) may be less dependable, altering demography. The fact that benthic communities even within pristine streams may be at risk when isolated within heavily impacted regions [68], and the tendency for upper reaches of Appalachian headwater streams to have resource extraction activities [69], warrants more attention to multi-dimensional wildlife community responses within aquatic-terrestrial linkages associated to shale gas development.

## Supporting information

S1 Table Annual shale gas disturbance activity at Lewis Wetzel Wildlife Management Area study streams in 2011, 2013, and 2014. Our research season for several ridgetop and stream research projects occurred relatively from April–July of each year, with time periods referring to this research season range. R = new ridgetop activity, S = new stream activity (streamside road activity or stream sedimentation), W = new well pad activity, P = parts considered disturbed from activity in previous years, N = no new activity. Superscripts B = Brief or intermittent activity period(s), E = Early in waterthrush breeding season, L = Late in waterthrush breeding season, and C = continuous activity.
(DOCX)

S2 Table Variables used to evaluate the demographic response and nest survival of Louisiana Waterthrush to aquatic prey and shale gas development. Nest survival is daily survival rate (DSR) over a 29-day nesting period. We evaluated Louisiana Waterthrush demographic response to aquatic prey and shale gas development using spatial generalized linear mixed models (SGLMMs).
(DOCX)

S1 Dataset Riparian habitat quality and aquatic prey (2013–2014). Data used to analyze the relationship between riparian habitat quality (EPA, HSI) and aquatic prey (EPT_RICHNESS, INTOLERANT_GENERA, GLIMPSS, WVSCI, BIOMASS, DENSITY) for years 2013–2014. Each year (YEAR) was analyzed separately while accounting for spatial (X, Y) and non-spatial (STREAM) sources of variability.
(XLSX)

S2 Dataset Riparian habitat quality and aquatic prey (2011). Data used to correlate riparian habitat quality (EPA, HSI) and aquatic prey (EPT_RICHNESS, INTOLERANT_GENERA, GLIMPSS, WVSCI, BIOMASS, DENSITY) for the year 2011. The 2011 study evaluated waterthrush demographic response to aquatic prey at a watershed-scale [29].
(TXT)

S3 Dataset Shale gas disturbance and aquatic prey. Data used to analyze the relationship between shale gas disturbance (TerrGas, TerrRunoff, NestGas) and aquatic prey (EPT_RICHNESS, INTOLERANT_GENERA, GLIMPSS, WVSCI, BIOMASS, DENSITY) for years 2013–2014. Each year (YEAR) was analyzed separately while accounting for spatial (X, Y) and non-spatial (STREAM) sources of variability.
(XLSX)

S4 Dataset Waterthrush demography and aquatic prey. Data used to analyze the relationship between waterthrush demography (CLUTCH, FLEDGLINGS, TERRITORY_LENGTH, TERRITORY_DENSITY) and aquatic prey (EPT_RICHNESS, INTOLERANT_GENERA, GLIMPSS, WVSCI, BIOMASS, DENSITY) for years 2013–2014. Each year (YEAR) was analyzed separately while accounting for spatial (X, Y) and non-spatial (STREAM) sources of variability.
(XLSX)

S5 Dataset Waterthrush nest survival and aquatic prey (2013). MARK-formatted file (.inp file extension) used to analyze the relationship between waterthrush nest survival and aquatic prey for the year 2013.
(INP)

S6 Dataset Waterthrush nest survival and aquatic prey (2014). MARK-formatted file (.inp file extension) used to analyze the relationship between waterthrush nest survival and aquatic prey for the year 2014.
(INP)
