# Supplementary material for: Demographic characteristics of an avian predator, Louisiana Waterthrush (Parkesia motacilla), in response to its aquatic prey in a Central Appalachian USA watershed impacted by shale gas development
Source: PLoS One. 2018 Nov 28;13(11):e0206077. doi: 10.1371/journal.pone.0206077 (PMC6261416; doi:10.1371/journal.pone.0206077)
Supplement: S1 Table — Our research season for several ridgetop and stream research projects occurred relatively from April–July of each year, with time periods referring to this research season range. R = new ridgetop activity, S = new stream activity (streamside road activity or stream sedimentation), W = new well pad activity, P = parts considered disturbed from activity in previous years, N = no new activity. Superscripts B = Brief or intermittent activity period(s), E = Early in waterthrush breeding season, L = Late in waterthrush breeding season, and C = continuous activity. (DOCX) [file pone.0206077.s001.docx]

S1 Table. Annual shale gas disturbance activity at Lewis Wetzel Wildlife Management Area study streams in 2011, 2013, and 2014. Our research season for several ridgetop and stream research projects occurred relatively from April–July of each year, with time periods referring to this research season range. R = new ridgetop activity, S = new stream activity (streamside road activity or stream sedimentation), W = new well pad activity, P = parts considered disturbed from activity in previous years, N = no new activity. Superscripts B = Brief or intermittent activity period(s), E = Early in waterthrush breeding season, L = Late in waterthrush breeding season, and C = continuous activity.

As a result of previous and newly started ridgetop activity in 2010–2011 the whole downstream network of some streams became at risk for sedimentation and surface runoff for the remainder of the study. In 2013, construction started on a new compressor station located before reaching Snake North, meaning all headwater stream bases emptying into Buffalo Run north of this site was near heavy truck traffic from 2013–2014. In late 2013 (June–July), construction started on a new well pad at the base of Owl Run which was previously a homestead with all drilling completed by July–August 2014. Well pad construction started on the ridgetops above Olive Run in 2013 with wells not becoming active until 2014; otherwise the stream remained mostly undisturbed except for maintenance of a forested track for pump jack accessibility. Activity at Buffalo East and West Run was mainly concentrated at their confluence where an active shale gas pad was located, especially in 2013–2014 where disturbance was otherwise minimal. In 2013–2014, Wyatt and especially Sees Run were subject to sedimentation slips into the stream from compromised erosional control below well pads or access roads, especially during any rainfall event; Wyatt Run had a new unsodded pipeline connector that caused brief sedimentation early in the research season.

In 2014, activity at Slabcamp Run increased from the previous year from a well pad being re-drilled but was commonly subject to streamside disturbance. Sees Run had increased sedimentation in 2014 from an active access road hillside partially collapsing and filling a stream valley. In summary, 2013 disturbances were just starting to occur but did not necessarily directly affect the streams during the time waterthrush were sampled in 2013, and in 2014 shale gas activity peaked again, particularly at Slabcamp Run, but did not achieve 2011 levels of activity.

| **Study Streams** | **2011** | **2013** | **2014** |
| --- | --- | --- | --- |
| Buffalo East Run | P, S^B^ | P, W^EB^ | P, W^B^ |
| Buffalo West Run | P, S^B^ | P, W^EB^ | P, W^B^ |
| Carpenter Run | N | N | N |
| Hiles Run | N | N | N |
| Huss Pen Run | N | N | N |
| Megans Run | S^B^, R^C^ | P, R^C^ | P, R^C^, W |
| Nettles Run | P, R^L^ | P, S^B^ | P |
| Olive Run | P, W, S | P, S^B^, R | P, S^B^, R, W |
| Owl Run | P, S | P, W^L^ | P, W^C^ |
| Sees Run | P, R^C^, W, S | P, R^C^, S^C^, W | P, R^C^, S^C^ |
| Slabcamp Run | P, W, S | P, W, S | P, R^L^, W^C^, S^C^ |
| Snake North Run | P, S | P | P |
| Snake South Run | P, S | P | P |
| Wyatt Run | R^C^, W | P, R^C^, S^EB^, W | P, R^C^, S^B^, W |
| **Nest/Stream Sampling Dates** | **May 6 – 9** | **May 22 – July 28** | **June 16 – July 6** |
