# Supplementary material for: Demographic characteristics of an avian predator, Louisiana Waterthrush (Parkesia motacilla), in response to its aquatic prey in a Central Appalachian USA watershed impacted by shale gas development
Source: PLoS One. 2018 Nov 28;13(11):e0206077. doi: 10.1371/journal.pone.0206077 (PMC6261416; doi:10.1371/journal.pone.0206077)
Supplement: S2 Table — Nest survival is daily survival rate (DSR) over a 29-day nesting period. We evaluated Louisiana Waterthrush demographic response to aquatic prey and shale gas development using spatial generalized linear mixed models (SGLMMs). (DOCX) [file pone.0206077.s002.docx]

S2 Table. Variables used to evaluate the demographic response and nest survival of Louisiana Waterthrush to aquatic prey and shale gas development. Nest survival is daily survival rate (DSR) over a 29-day nesting period. We evaluated Louisiana Waterthrush demographic response to aquatic prey and shale gas development using spatial generalized linear mixed models (SGLMMs).

| **Variable of interest** | **Notation** | **Analysis** |
| --- | --- | --- |
| Nest Age | NestAge | Nest Survival |
| Time within-season (quadratic time trend) | TT | Nest Survival |
| Average Daily Rainfall | Rain | Nest Survival |
| Percent of Stream Disturbed by Shale Gas | StreamGas | Description only |
| Percent of Stream with Potential Contaminant Runoff | StreamRunoff | Description only |
| Shale Gas Nest Disturbance  (undisturbed=0, disturbed=1) | NestGas | SGLMM |
| Percent of Territory Disturbed by Shale Gas | TerrGas | SGLMM |
| Percent of Territory with Potential Contaminant Runoff | TerrRunoff | Nest Survival, SGLMM |
| Clutch Size | Clutch Size | SGLMM |
| Number of Fledglings | Fledglings | SGLMM |
| Territory Length | Territory Length | SGLMM |
| Territory Density | Territory Density | SGLMM |
| West Virginia Stream Condition Index | WVSCI | Nest Survival, SGLMM |
| Genus Level Index of Most Probable Stream Status | GLIMPSS | Nest Survival, SGLMM |
| Aquatic Prey Biomass | Biomass | Nest Survival, SGLMM |
| Aquatic Prey Density | Density | Nest Survival, SGLMM |
| Number of Intolerant Genera | Intolerant Genera | Nest Survival, SGLMM |
| Ephemeroptera, Plecoptera, Trichoptera (EPT) Richness | EPT Richness | Nest Survival, SGLMM |
| Habitat Suitability Index (HSI) score | HSI | SGLMM |
| Environmental Protection Agency (EPA) Rapid Bioassessment index | EPA | SGLMM |
